# Effectiveness of Iron Therapy on Breath Holding Spells in the Children

**Published:** 2019

**Authors:** Elham BIDABADI, Sedigheh POORNABI DARZI, Parham MASHOUF, Tamkin SHAHRAKI

**Affiliations:** 1 Pediatric Diseases Research Center, Guilan University of Medical Sciences, Rasht, Iran

**Keywords:** Breath holding spells, Iron therapy, Child

## Abstract

**Objectives:**

The pathophysiology and mechanism of Breath-Holding Spells (BHS) remain controversial, and the relationship between BHS and anemia has not been clarified, although iron supplementation appears to be effective in many patients. We aimed to assess the probable relation of iron level with initiation of these spells in children.

**Materials & Methods:**

Overall, 42 children with a diagnosis of BHS, aged between 6 months to 2 yr were enrolled during Mar 2015 to Dec 2016 at Rasht 17th Shahrivar Hospital, Rasht, northern Iran. Ferrous sulfate solution prescribed 6 mg/kg/d, 3 times daily, for all of cases, regardless of their iron levels, and the response to the treatment was evaluated.

**Results:**

Twenty-five patients were boys (59.52%). The mean age for all patients was 11.71±4.63 months. Positive family history detected in 33.33%; iron deficiency anemia in 21.42%, depletion of iron stores in 52.38%, and normal iron status in 26.19% of cases. Simple spells showed significantly higher mean of Hb in comparison with severe spells (*P*=0.008); also increased number of spells per month significantly decreased the mean of Hb (*P*=0.007). Mean frequency of spells was 40.14±47.08 before and 11.14±31.10 after iron therapy, per month (*P*<0.0001). Overall, 32 patients (76.19%) had complete control of spells, 7 patients (16.66%) partial, 2 cases (4.76%) weak, and 1 child (2.38%) no response after iron therapy.

**Conclusion:**

Iron deficiency anemia may have an important role in BHS, and treatment of anemia may decrease number of the spells.

## Introduction

“Possibly the earliest report of Breath-Holding Spells (BHS) was published in 1737 by Nicolas Culpepper” (1-4).

Episodes of BHS are usually accrued in children without any underlying diseases, during expiration. These attacks are scary for parents because they are similar to some life-threatening diseases. About 60% of affected children have the cyanotic form of BHS, about 20% of affected children have the pallid form, and additional 20% have both types of breath holding spells. The family history is 20%-30% positive in these patients. Pathophysiology and mechanism of BHS remain controversial (5-15).

Iron deficiency anemia is frequently seen in infants (16,17). Iron is very important for neurological functioning, including neurotransmitters, myelin formation, and brain energy metabolism (18-23). As iron is a very important factor for proper neurological functions, it may have an important role in initiation of BHS. Iron supplements appear to be effective in many BHS patients (24-27).

We aimed to assess the probable relation of iron level with initiation of these spells in children.

## Materials & Methods

All 6 months to 2 yr children with diagnosis of BHS, without history of previous convulsion, developmental delay, neurological deficit, chronic illness, primary cardiac disease, current treatment with anticonvulsant medications, or not reliable for drug consumption were enrolled, conducted during Mar 2015 to Dec 2016 at Rasht 17th Shahrivar Hospital, Rasht, Iran. Demographic information, as well as data about developmental milestones, type of BHS, age at onset, frequency and duration and severity of attacks before and after iron therapy, associated phenomenon, usual triggering factors, history of previous iron therapy, positive family history, and overall response to treatment were collected for all cases.

Patients attacks were classified into 3 types: cyanotic, pallid, and mixed, based on skin color change during attacks. These spells were classified as simple and severe (with loss of consciousness and jerking movements) episodes.

Routine hematological tests were performed for all patients on the early morning. Red blood cell count, hemoglobin (Hb), and hematocrit (Hct), as well as mean corpuscular volume (MCV), mean corpuscular hemoglobin (MCH), mean corpuscular hemoglobin concentration (MCHC), total iron binding capacity (TIBC), plasma ferritin (PF), and serum iron concentration, were collected for each patient.

The patients were divided into 3 groups, based on hematological status including normal serum iron, depletion of iron stores (decreased serum iron and serum ferritin, increased TIBC, but normal Hb, MCV, and MCH), and iron deficiency anemia. We defined iron deficiency anemia as follows: Hb less than 10.5 g% (for 6 months to 2 yr old cases), Hct<33%, MCV less than 70 fl, MCH less than 23 pg, MCHC less than 30 g/%, RBC<3.7, and serum iron concentration less than 40 µg/dl (for patients younger than 12 months old) and greater than 50 µg/dl (for cases older than 12 months old), PF less than 7 µg/I, and TIBC greater than 430 mcg/dl.

We prescribed oral ferrous sulfate solution 6 mg/kg/d, 3 times daily, for all of cases, regardless of their iron levels. We evaluated the clinical condition of the patient with 3 months follow up; their response to treatment defined as follows: "complete", the attacks disappeared completely; "partial", >50% reduction in the number of attacks; “weak", 10%-50% reduction in the attacks; and "no response", <10% reduction in the attacks.

Written informed consent was obtained for all parents. This study was approved by the Ethics Committee at Guilan University of Medical Sciences (GUMS).

Variables are shown by mean ± SD and compared by means of the paired *t* test, ANOVA, and McNemar test. Statistical significance was calculated as *P*<0.05.

## Results

**Figure 1:**
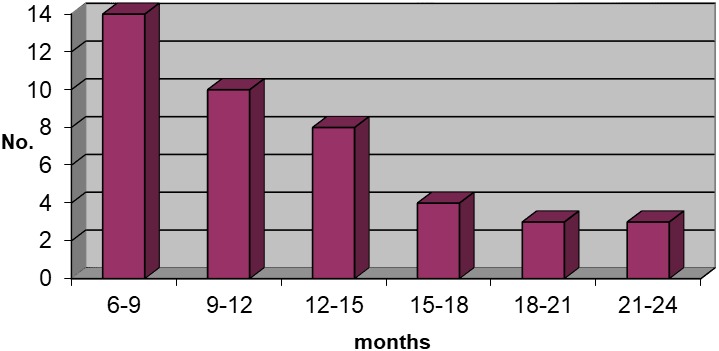
Distribution of patients between age groups

There were 42 children with diagnosis of BHS, aged between 6 months to 2 yr (mean age was 11.71±4.63 months), 25 patients were boys (59.52%) and 17 were girls (40.48%), with male predominance in the all age groups (boy/girl ratio was 1.47:1) (Figure 1). 

Cyanotic spells were observed in 35 patients [(83.33%), 22 boys and 13 girls). Pallid spells in 4 patients [(9.52%), 1 boy and 3 girls] and mixed spells in 3 cases [(7.14%), 2 boys and 1 girl]. Positive family history of BHS was seen in 14 children (33.33%). While iron deficiency anemia was observed in 9 children (21.42%), 22 children (52.38%) had depletion of iron stores of the body, and 11 cases (26.19%) had normal iron status.

Fifteen patients (35.71%) had history of previous iron supplement therapy [one patient (6.66%) with iron deficiency anemia, 5 cases (33.33%) with depletion of iron stores of the body, and 9 patients (60%) with normal iron status].

Patient's iron level was shown in Table 1. Simple spells were shown significantly higher mean of Hb in comparison with severe spells (*P*= 0.008); also increased number of spells per month significantly decreased the mean of Hb (*P*= 0.007).

Results of iron therapy in children with BHS are detailed in Table 2. Overall, 32 patients (76.19%) had complete control of spells, 7 patients (16.66%) partial, 2 cases (4.76%) weak, and 1 child (2.38%) no response after iron therapy.

**Table 1:** Patient’s Blood indices

	**Mean of Hb (g%)**	**Iron deficiency anemia**	**Depletion of iron stores**	**Normal iron**	***P*** **-value**
**30<** **spells/month** **(No.=13) **	**10.95±0.51**	5 (38.46%)	7 (53.84%)	1 (7.69%)	0.007
**15-30 spells/month** **(No.=12)**	**11.45±0.62**	2 (16.66%)	9 (75%)	1 (8.33%)	
**15≥** **spells/month** **(No.=17)**	**11.55±0.65**	2 (11.76%)	6 (35.29%)	9 (52.94%)	
**Simple spells** **(No.=29)**	**11.63±0.71**	6 (21%)	19 (65.5%)	4 (13.5%)	0.008
**Severe spells** **(No.=13)**	**10.67±0.48**	11 (84.5%)	2 (15.5%)	0 (0%)	

**Table 2:** Characteristics of spells before and after iron therapy

**Characteristic of spells**		**Before iron therapy**		**After iron therapy**		***P-*** **value**
		**No.**	**%**	**No.**	**%**	
Frequency /month	>30	13	30.95	4	9.52	<0.0001
	15-30	12	28.57	1	2.38	
	<15	17	40.47	37	88.09	
	mean	40.14 ± 47.08		11.14 ± 31.10		
Severity	Simple	29	69.04	41	97.61	<0.001
	Severe	13	30.95	1	2.38	

**Table 3:** Summery of studies

**Citation**	**Study group**	**Study type**	**Result**
Bhatia et al (1990) (45)	50 children with BHS	Case series	96% of the study group had iron deficiency anemia. 82% showed Response within 2 wk. After three weeks 100% of cases had shown an improvement.
Colina and abelson (1995) (15)	2 cases	Case report	Improvement after treatment of anemia
Daoud et al (1997) (43)	67 children with BHS	Clinical trial With ferrous sulfate or placebo	Complete response in 51.5% of treated versus 0% in non-treatment group. Partial response in 36.4% of treated group versus 5.9% in non-treatment group.
Mocan et al (1999) (44)	91 children with BHS	Case-control	Complete response in 50.7% and Partial response in 33.3% and no response in 16% after therapy.
Zubcevich et al (2000) (47)	17 children with BHS	Before & after study	Anemia was present in 76% of cases. Improvement in 88.9% after iron therapy.
Mul et al (2005) (46)	1 case	Case report	Improvement after treatment of anemia

## Discussion

The present study designed to assess the probable relation of iron level with BHS in children, and findings revealed that iron deficiency plays an important role on initiation of these spells. Results of this study had some similarities with other studies in this field, as well as some differences. 

Linder in a prospective study on 697 patients reported mild BHS in 4.7% and severe BHS in 1.7% of cases (5), consistent with data presented in the current report.

Positive family history was reported in 20%-30% of BHS patients; genetic factors may play an important role in this disease, with a low-penetrance autosomal dominant factor (28). Although about 50% positive family history reported in some studies (24); but some other studies failed to demonstrate positive family history in the BHS patients (29). We revealed 33.33% positive family history in our patients, confirmed the results of the first studies.

Multiple spells per week reported in majority of patients; about one-third of cases will experience more than one spell per day (30), similar to our cases.

Cyanotic spells were seen in 54% of cases, pallid in 22%, both types in 12%, and not classifiable in 7% of patients (31, 32). This is different from our cases, as the cyanotic: pallid: mixed ratio in our patients has emerged as 35:4:3.

Iron deficiency anemia is common worldwide, especially in children living in developing countries (21, 33). Several causative factors (psychological problems and different physiologic mechanisms, such as cardiac inhibition through vagus nerve with painful stimuli, or central inhibition of respiratory movements, role of decreased sensitivity of CNS to hypoxia and hypercapnia, abnormalities in lung reflexes and movements) for BHS are suggested, but its exact etiology is unclear (6, 7). Iron deficiency anemia is frequently seen in BHS patients, and iron therapy may have a role in its treatment (Table 3). Holowach et al first described the association between BHS and anemia (26).

The causative mechanism of iron deficiency in BHS is not fully understood, probably it plays a role through neurotransmitters, enzymes, and catecholamine metabolism (31). Some other studies suggested the role of reduced oxygen availability to CNS, because of adverse effects of anemia on oxygen uptake in the lungs (24), and the probable role of erythropoietin, nitric oxide and interleukin 1 (25). Our study revealed the important role of iron in the treatment of BHS because the mean frequency of spells was significantly higher before iron therapy (*P*<0.0001); and severity of spells significantly decreased after therapy (*P*<0.001). We observed complete control of BHS in 76.19% of our cases, after iron therapy. In our cases, simple spells are shown significantly higher mean of Hb in comparison with severe cases; Mean Hb level was significantly lower when number of spells per month increased.

**In conclusion,** iron deficiency anemia might have an important role in BHS, and treatment of anemia may decrease number of the spells.

## References

[1] Meigs JF (1848). A Practical Treatise on the Diseases of Children.

[2] Neumann H (1905). Ueber das wegbleiben kleiner kinder. Archiv. Kinderheilk.

[3] Ibrahim J (1911). Ueber respiratorische affektkrampfe im fruhen kindesalter (das sogenannte “wegbleiben” der kinder). Zeitschrift fur das gesamte Fortsch. Neurol. Psychiat.

[4] Stier E (1920). Ueber ohnmachtsanfalle, besonders bei kindern. Deutsch Med Wschr.

[5] Linder CW (1968). Breath holding spells in children. Clin Pediatr.

[6] Lombroso CT, Lerman P (1967). Breathholding spells (cyanotic and pallid infantile syncope). Pediatrics.

[7] DiMario FJ Jr (1992). Breath-holding spells in childhood. Am J Dis Child.

[8] Bridge EM, Livingston S, Tietze C (1943). Breath-holding spells: their relationship to syncope, convulsions, and other phenomena. J Pediatr.

[9] Evans OB (1997). Breath-holding spells. Pediatric Annals.

[10] DiMario FJ Jr, Burleson JA (1993). Behavior profile of children with severe breath-holding spells. J Pediatr.

[11] Colina KF, Abelson HT (1995). Resolution of breath-holding spells with treatment of concomitant anaemia. J Pediatr.

[12] DiMario FJ Jr, Chee CM, Berman PH (1990). Pallid breath holding spells, Evaluation of the autonomic nervous system. Clin Pediatr.

[13] DiMario FJ Jr, Bauer L, Volpe J, Baxter D (1997). Respiratory sinus arrhythmia in children with severe cyanotic breath holding spells. J Child Neurol.

[14] Kohyama J, Hasegawa T, Shimohira M, Fukumizu M, Iwakawa Y (2000). Rapid eye movement sleep in breath holders. J Child Neurol.

[15] Kahn A, Rebuffat E, Sottiaux M, Muller MF, Bochner A, Grosswasser J (1990). Brief airway obstruction during sleep in infants with BHS. J Pediatr.

[16] Florentino RF, Guirriec RM, Stekel A (1984). Prevalence of nutritional anemia in infancy and childhood with emphasis on developing countries. Iron nutrition in infancy and childhood.

[17] Stoltzfus R (2001). Defining iron-deficiency anemia in public health terms: a time for reflection. J Nutr.

[18] Lozoff B, Klein NK, Nelson EC, McClish DK, Manuel M, Chacon ME (1998). Behavior of Infants with Iron-Deficiency Anemia. Child Development.

[19] Rouault TA, Cooperman S (2006). Brain Iron Metabolism. Semin Pediatr Neurol.

[20] Youdim MBH, Ben-Shachar D, Yehuda S (1989). Putative biological mechanisms of the effect of iron deficiency on brain biochemistry and behavior. Am J Clin Nutr.

[21] Larkin EC, Rao GA, Dobbing J (1990). Importance of fetal and neonatal iron: adequacy for normal development of central nervous system. Brain, behavior and iron in the infant diet.

[22] Erikson KM, Jones BC, Hess EJ, Zhang Q, Beard JL (2001). Iron deficiency decreases dopamine D1 and D2 receptors in rat brain. Pharmacol Biochem Behav.

[23] Lozoff B, Georgieff MK (2006). Iron Deficiency and Brain Development. Semin Pediatr Neurol.

[24] Daoud AS, Batieha A, Al-Sheyyab M, Abuekteish F, Hijazi S (1997). Effectiveness of iron therapy on breath-holding spells. J Pediatr.

[25] Mocan H, Yildiran A, Orhan F, Erduran E (1999). Breath holding spells in 91 children and response to treatment with iron. Arch Dis Child.

[26] Holowach J, Thurston DL (1963). Breath-holding spells and anemia. N Engl J Med.

[27] Yilmaz S, Kükner C (1996). Anemia in children with breath-holding spells. J Pediatr.

[28] DiMario FJ Jr, Sarfarazi M (1997). Family pedigree analysis of children with severe breath-holding spells. J Pediatr.

[29] Hüdaoglu O, Dirik E, Yiş U, Kurul S (2006). Parental attitude of mothers, iron deficiency anemia, and breath-holding spells. Pediatr Neurol.

[30] DiMario FJ Jr (2001). Prospective Study of Children with Cyanotic and Pallid Breath-Holding Spells. Pediatrics.

[31] Oski FA, Honig AS (1978). The effects on the developmental scores of iron-deficient infants. J Pediatr.

[32] Laxdal T, Gomez MR, Reiher J (1969). Cyanotic and pallid syncopal attacks in children (breath-holding spells). Dev Med Child Neurol.

[33] Stoltzfus RJ, Mullany L, Black RE, Ezzati M, Lopez AD, Rodgers A (2004). Iron deficiency anemia. Comparative Quantification of Health Risks: Global and Regional Burden of Disease Attributable to Selected Major Risk Factors.

